# Intranasal dantrolene nanoparticles abolished depression behavior and memory loss as a disease‐modifying drug in 5XFAD mice

**DOI:** 10.1002/alz.089764

**Published:** 2025-01-09

**Authors:** Piplu Bhuiyan, Wenjia Zhang, Rebecca Chae, Kyelee Kim, Lauren St. Louis, Ying Wang, Ge Liang, Huafeng Wei

**Affiliations:** ^1^ Department of Anesthesiology and Critical Care, Perelman School of Medicine, University of Pennsylvania, Philadelphia, PA 19104, U.S.A., Philadelphia, PA USA

## Abstract

**Background:**

The vicious cycle between depression and dementia increases the risk of Alzheimer’s Disease (AD) pathogenesis and pathology. This study investigates therapeutic effectiveness versus side effects and the underlying mechanisms of intranasal dantrolene nanoparticles (IDNs) to treat depression behavior and memory loss in 5XFAD mice.

**Method:**

5XFAD and wild‐type B6SJLF1/J mice were treated with IDNs (IDN, 5 mg/kg) in Ryanodex formulation for a duration of 12 weeks. Early treatment groups (ETG) began treatment at 2 months until 5 months of age, and late treatment groups (LTG) began treatment at 9 months until 12 months of age. Behavioral assessments including depression (Tail suspension, open field), cognition (fear conditioning, Y‐ maze), olfaction (buried food test), and motor function (rotarod) were conducted between the ages of 5‐6 months in ETG and 12‐13 months in LTG. Blood and brain samples were obtained at 7 and 13 months in ETG and LTG, respectively. Blood Alanine transaminase measured by ELISA for liver function. The changes of brain proteins on Ca^2+^ release channels on the ER membrane (RyR‐2 and InsP_3_R‐1), MDA, pyroptosis regulatory proteins (NLRP3, Caspase‐1, full length or NT of Gasdermin D, cytotoxic (IL‐1β, IL‐18, IL‐6, TNF‐a) and cytoprotective (IL‐10) cytokines and PSD‐95, Synapsin‐1 determined using immunoblotting. Mice weight was measured monthly.

**Result:**

Compared to WT mice, there were significant depression behavior and memory loss in both ETG and LTG 5XFAD mice, which were abolished by IDNs. IDNs also significantly inhibited the pathologically increased proteins expression of RyR‐2 and InsP_3_R‐1, MDA‐modified proteins, pyroptosis regulatory proteins (NLRP3, Caspase‐1, NT‐GSDMD), neurotoxic cytokines (IL‐1β, IL‐18, IL‐6, TNF‐α). IDNs also increased the neuroprotective cytokines (IL‐10) and synaptic proteins (PSD‐95, Synapsin‐1) in the LTG mice. IDNs did not affect blood liver function enzymes, motor or smell function, body weight in both ETG and LTG WT or 5XFAD mice.

**Conclusion:**

IDNs robustly protect both depression behavior and memory loss in young and aged 5XFAD mice, with no adverse effects with chronic use. This was associated with its potent inhibition of inflammation, pyroptosis, and associated synapse protein loss. IDNs emerge as a promising therapeutic intervention for both depression and dementia, particularly in AD.

